# TDP-43-mediated amyotrophic lateral sclerosis: new/hidden insights from *Drosophila*

**DOI:** 10.3389/fcell.2025.1677090

**Published:** 2025-10-16

**Authors:** Davide Colaianni, Nadia Ceccato, Pietro Antolini, Carmela Conte, Cristiano De Pittà, Fabian Feiguin, Gabriella M. Mazzotta

**Affiliations:** ^1^ Department of Biology, University of Padova, Padova, Italy; ^2^ Department of Pharmaceutical Sciences, University of Perugia, Perugia, Italy; ^3^ Department of Life and Environmental Sciences, University of Cagliari, Cagliari, Italy

**Keywords:** TDP-43, amyotrophic lateral sclerosis, *Drosophila melanogaster*, TBPH, gene expression

## Abstract

Transactive response DNA-binding protein 43 (TDP-43) is a key factor in motor neurons and related neurodegenerative disorders, and the presence of cytoplasmic aggregates of TDP-43 is a major hallmark of diseases such amyotrophic lateral sclerosis (ALS) and frontotemporal lobar degeneration (FTLD). Nevertheless, little is known about early developmental effects or the systemic nature of TDP-43-mediated pathology. *Drosophila melanogaster* is acknowledged as a powerful genetic model for studying the genetic inheritance and the behavioral and developmental processes associated with human neurodegenerative diseases, including ALS. To better understand the possible roles and potential pathogenic mechanisms of TDP-43 protein in the pathogenesis of ALS, we performed a transcriptomic analysis of larvae from a *Drosophila* model knock-out (KO) for the *TBPH* gene, the fly *TDP-43* ortholog. Interestingly, the Gene Ontology (GO) analysis highlighted some pathways not yet associated with this pathology and this model. We identified several genes encoding for serine proteases, a class of enzymes that in the central nervous system (CNS) play important roles in neural development, synaptic plasticity, and neurodegeneration. Our work provides insights into novel pathological mechanisms underlying the disease, thereby opening new pathways for drug discovery.

## Introduction

The *TDP-43* gene (*TAR DNA-binding protein 43*) has been implicated in both sporadic and familial forms of amyotrophic lateral sclerosis (ALS) and frontotemporal dementia (FTD) (de Boer et al., 2020). The human TDP-43 protein is composed of 414 amino acids and includes several functional domains: an N-terminal domain, a nuclear localization sequence (NLS), two RNA recognition motifs (RRM1 and RRM2) essential for RNA binding, and a C-terminal glycine-rich domain (Mompeán et al., 2016). TDP-43 is a member of the heterogeneous nuclear ribonucleoproteins (hnRNPs) family and plays a key role in RNA metabolism, including splicing, stability, and transport. The protein shuttles between the nucleus and the cytoplasm *via* both active and passive mechanisms (Pinarbasi et al., 2018). Under physiological conditions, particularly in motor neurons, TDP-43 is predominantly localized in the nucleus, with only low levels found in the cytoplasm and cytoplasmic organelles such as mitochondria (Wang et al., 2016). Mutations in the *TDP-43* gene account for approximately 4%–5% of familial ALS cases and around 1% of sporadic ALS cases and are typically inherited in an autosomal dominant manner (Alsultan et al., 2016). In most individuals with ALS carrying *TDP-43* mutations, the protein forms cytoplasmic aggregates, which represent the principal component of ubiquitinated inclusions found in both neurons and glial cells (Tan et al., 2017). Although the exact pathogenic mechanisms by which TDP-43 contributes to neurodegeneration remain unclear, growing evidence points to disruptions in several RNA-related cellular processes as potential contributors to neuronal death. Current research supports two main, non-mutually exclusive mechanisms of TDP-43 toxicity: a nuclear loss-of-function and a cytoplasmic gain-of-function. These involve the depletion of TDP-43 from the nucleus - where it normally regulates RNA metabolism - and its mislocalization and accumulation in the cytoplasm, where it becomes sequestered into insoluble inclusions (Cascella et al., 2016). This cytoplasmic aggregation is believed to induce toxic effects and cellular damage, playing a central role in ALS pathogenesis (Prasad et al., 2019). Overall, while TDP-43-mediated ALS and ALS in general predominantly affects motor neurons, it is nowadays widely recognized as a systemic disease, involving disruption of multiple molecular mechanisms in different tissues (Moresi, 2023; Appel et al., 2021).

The fruit fly *Drosophila melanogaster* has long been established as a great tool to study neurodegenerative diseases (Marsh and Thompson, 2006) including ALS (Hegde and Srivastava, 2022), and a series of transgenic and knock-out models of TDP-43-mediated ALS have been generated and characterized (Romano et al., 2012). In particular, one of the first generated and well-characterized TDP-43-mediated ALS *Drosophila* models is the *TBPH* ^Δ23/Δ23^ fly (Feiguin et al., 2009). *TBPH* is the *Drosophila TDP-43* ortholog, broadly conserved in terms of both structure and functions (Romano et al., 2012; Diaper et al., 2013). This model is characterized by a 1,616 bp deletion that partially removed *TBPH* coding and regulatory regions, resulting in the complete abolishment of endogenous TBPH protein expression (Feiguin et al., 2009). Hence, this strain exhibits high lethality and extremely reduced lifespan, with dramatic locomotor impairment both in the adult and in the larva (Feiguin et al., 2009). Moreover, at a cellular level, *TBPH* ^Δ23/Δ23^ larvae display morphological defects in the presynaptic terminals of motoneurons at the neuromuscular junctions, with reduced number of axonal branches and synaptic boutons present inside the muscles (Feiguin et al., 2009). Overall, this model has been proven to fully recapitulate TDP-43-mediated ALS pathology, exhibiting significant similarities with mice models (Gendron and Petrucelli, 2011) and patients, and over the subsequent years it has been used to further investigate different aspects of the disease, in particular TDP-43-regulated genes and their roles in the pathogenesis and pathophysiology of ALS (Godena et al., 2011; Miskiewicz et al., 2014; Langellotti et al., 2018; Romano et al., 2020; Strah et al., 2020; Romano et al., 2021). However, a comprehensive molecular characterization of *TBPH* ^Δ23/Δ23^ model at the gene expression level in developmental stages was still lacking. Given the aim of investigating alterations occurring already from the early stages of development, we chose to focus our attention on third instar larvae. Moreover, considering the systemic nature of the disease, we decided to analyse the gene expression profile based on RNA extracted from the entire larval body. Therefore, in this work, we provide the first molecular characterization of whole third instar *TBPH* ^Δ23/Δ23^ larvae, through which we not only validated its relevance as a TDP-43/TBPH-mediated ALS model even at the transcriptomic level, but we also uncovered novel and hidden pathological mechanisms underlying the disease, thereby opening new pathways for drug discovery.

## Methods

### Fly strains

*w* ^*1118*^ (control) and *TBPH* ^Δ23^/*CyO* (BDSC #93599) (Feiguin et al., 2009) *D*. *melanogaster* strains were used. The latter was then crossed with a fly strain carrying a balancer chromosome *CyO* associated with a GFP sequence, to obtain the *TBPH* ^Δ23^/*CyO*-*GFP* strain allowing for the selection of *TBPH* ^Δ23/Δ23^ (hereinafter referred to as *TBPH* knock-out) larvae. Flies were raised at 23 °C under a 12:12 h light-dark (LD) cycle and fed on a standard cornmeal-yeast agar food.

### RNA extraction

For each sample, 10 third instar larvae of the selected genotype were collected and frozen in liquid nitrogen. Total RNA was extracted by using the TripleXtractor reagent (GRiSP Research Solutions, Porto, Portugal) according to the manufacturer’s instructions. RNA concentration was measured using the NanoDrop 2000c spectrophotometer (Thermo Fisher Scientific, Waltham, USA) and RNA integrity was assessed by electrophoresis using the Agilent 4,150 TapeStation (Agilent Technologies, Santa Clara, USA). Only samples with an RNA Integrity Number (R.I.N.) value higher than 8.0 were used for gene expression analysis.

### RNA-seq and gene ontology (GO) analyses

The RNA-seq analysis was conducted on three independent samples of RNA extracted from *TBPH* ^Δ23/Δ23^ and *w* ^*1118*^ whole third instar larvae. The experiment was performed by the NGS Facility (Department of Biology, University of Padova, Padova, Italy). cDNA libraries were constructed from 450 ng of total RNA by using the QuantSeq 3′ mRNA-Seq Library Prep Kit for Illumina (FWD) (Lexogen, Vienna, Austria) according to the manufacturer’s instructions. The workflow consists of first strand cDNA synthesis with oligo (dT) primers containing an Illumina-compatible sequence at the 5′ end, RNA template removal, second strand synthesis with random primers containing an Illumina-compatible linker sequence at the 5′ end, purification using magnetic beads to remove all reaction components, and PCR amplification to add the complete adapter sequences and to generate the final library. The libraries were quantified with the Qubit Flex Fluorometer (Invitrogen, Carlsbad, USA) and quality tested by Agilent 4150 TapeStation system (Agilent Technologies, Santa Clara, USA). Sequencing was carried out in single-end mode (150 bp) by using NovaSeq X Plus (Illumina, San Diego, USA) with a targeted sequencing depth of 30 million reads per sample. Base-calling was performed using RTA2 software (Illumina, San Diego, USA). File conversion and demultiplexing were performed using bcl2fastq software (version 2.20.0). Sequence reads are available on NCBI BioProject database with the accession number PRJNA1289126. Raw reads were trimmed to remove adapter sequences using cutadapt (version 4.9). In accordance with the guidelines provided by Lexogen, the poly(A) tails and the first 12 bases of each read were trimmed. Additionally, the --nextseq-trim = 10 parameter was applied to account for quality score bias associated with Illumina’s two-color chemistry. The abundances of all *D*. *melanogaster* transcripts annotated by ENSEMBL (release 112) were estimated using the Salmon software (version 1.10.3) (Patro et al., 2017) with --noLengthCorrection option enabled and then summarized at the gene level using tximport (version 1.32.0) (Soneson et al., 2015). Genes were filtered by their expression levels using the strategy described in Chen et al. (2016), as implemented in the edgeR package (version 4.2.1) (Robinson et al., 2010) with default parameters. A total of 11,328 genes were retained. Gene-level counts were normalized using the TMM method (edgeR, version 4.2.1) and for unwanted variation using EDASeq (version 2.38.0) and RUVSeq (version 1.38.0; RUVg method, k = 1 confounding factors) (Risso et al., 2014). Differential expression was tested with edgeR (version 4.2.1) using a GLM model. Genes with an adjusted p-value (FDR) <0.05 after correction for multiple testing (Benjiamini-Hochberg method) were considered differentially expressed (Supplementary Table S1). All the heatmaps were obtained using the Morpheus software (https://software.broadinstitute.org/morpheus, Broad Institute, USA). Finally, to investigate the molecular functions of the differentially expressed genes and the biological processes in which they were involved, a Gene Ontology (GO) functional enrichment analysis was performed using ShinyGO (Ge et al., 2020), applying a false discovery rate (FDR) <0.05 (Supplementary Table S2).

### Analysis of gene tissue distribution

The tissue distribution of the genes of interest was assessed on FlyAtlas 2 (flyatlas2.org) (Krause et al., 2022). For each gene, the tissue with the highest scores in terms of Larval FPKM and Enrichment was identified as the main tissue of expression; genes with no expression or equal scores were included in the “Not Assigned/N.A.” group. All analysed genes and their FlyAtlas 2 Larval FPKM and Enrichment scores in each tissue are reported in Supplementary Table S3.

### Differential transcript usage (DTU) analysis

Differential Transcript Usage (DTU) analysis was carried out following the workflow described by Love et al. (2018), using transcript-level quantification from Salmon. Transcript counts were imported with tximport version 1.32.0 (Soneson et al., 2015) using countsFromAbundance = “no”, appropriate for 3′RNA-seq data where transcript length bias is minimal. Transcript-to-gene annotations were generated from the Ensembl *Drosophila melanogaster* GTF (release BP46.112) using the GenomicFeatures package version 1.56.0 (Lawrence et al., 2013). Filtering and model fitting were performed with DRIMSeq version 1.32.0 (Nowicka and Robinson, 2016). We kept transcripts that met three conditions: first, they appeared at least 5 times in 3 or more samples; second, they made up 5% or more of their gene’s transcripts in at least 2 samples; and third, their associated gene was present at least 10 times across 4 or more samples. DRIMSeq’s Dirichlet-multinomial model was used to estimate transcript usage and test for differential usage between conditions. Gene- and transcript-level p-values were corrected using stageR version 1.26.0 (Van den Berge et al., 2017) to control the overall false discovery rate (target 0.05). Following guidance from the original study, NA p-values from DRIMSeq were replaced with 1, before the stageR step, to allow stage-wise testing. Genes that passed the stageR screen were considered showing DTU between control and *TBPH* KO. Limitations affecting sensitivity and comprehensiveness could be identified for the DTU analysis. The filtering steps, while essential for model stability and false positive control, remove low-abundance isoforms, potentially excluding biologically relevant events. Moreover, using 3′ RNA-seq protocols provides limited coverage of full-length transcripts and may fail to detect isoforms with differential usage occurring outside the 3′region or those with poor 3′end capture efficiency. Finally, the DRIMSeq statistical framework, though designed for robust differential proportion testing, employs conservative multiple testing correction procedures that prioritize specificity over sensitivity.

## Results

### Gene expression signature of *TDP-43*/*TBPH* knock-out larvae

We extracted total RNA from 3 *TBPH* knock-out (KO) and 3 *w* ^*1118*^ (CTR) whole larvae samples and performed an RNA-seq experiment on the three biological replicates per genotype. As indicated by the Principal Component Analysis (PCA) (Supplementary Figure S1), the two populations clustered distinctly and exhibited a clear separation, highlighting a markedly different gene expression profile. As a result, a large number of differentially expressed genes (DEGs) were identified between the two populations. Specifically, out of a total of 1718 DEGs (identified at FDR <0.05), 845 and 873 genes were found to be respectively down- and upregulated in *TBPH* KO vs. control larvae (Supplementary Figure S2; Supplementary Table S1).

### GO analysis highlights established and emerging paradigms in ALS pathology

**FIGURE 1:**
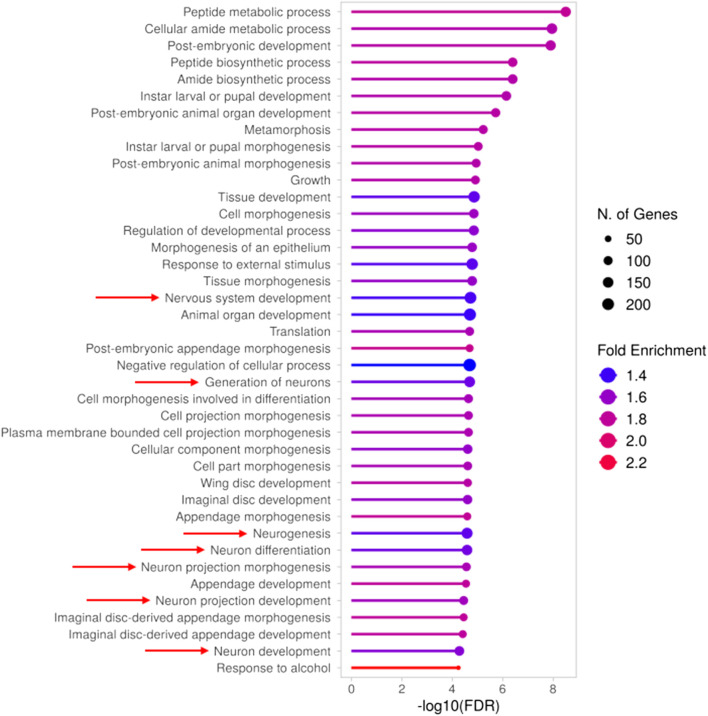
Gene Ontology (GO) analysis (BP) of differentially expressed genes in *TBPH* KO larvae. Results obtained from the GO analysis performed on the genes found to be differentially expressed between *TBPH* KO and control larvae. The top 40 significantly enriched biological processes (BP) are depicted, with red arrows indicating those associated with neuronal development and morphogenesis. The complete list of enriched terms is provided in Supplementary Table S2.

To deepen our molecular characterization of the *TBPH* KO larval model, starting from the 1718 DEGs we performed a GO analysis to investigate their molecular functions and the biological processes in which they were involved (Supplementary Table S2). As expected, given the ongoing development of the larvae, most of the identified Biological Processes (BP) terms were broadly associated with developmental processes (Figure 1). However, among them, we identified a substantial number of terms specifically related to neuronal development, morphogenesis, and physiology (Figure 1, indicated by red arrows), and in particular to neuronal projections (Supplementary Table S2), which suggests a neurodegenerative phenotype associated with ALS pathology. Indeed, the altered expression levels of several of the genes taking part in the enriched neuronal processes were previously associated with ALS and in general with neurodegeneration. As a matter of example, we reported the downregulation of *Atx2* and *Nedd4*, together with the upregulation of key drivers of ALS such as *Sod1* (identified at FDR <0.1) and *CHMP2B*, all of which are discussed in more detail in the final section. Altogether, the gene expression profile of *TBPH* KO larvae is consistent with that expected under such pathological conditions, thereby validating and further supporting its relevance as a *TDP-43*/*TBPH*-mediated ALS model, even at the transcriptomic level.

**FIGURE 2:**
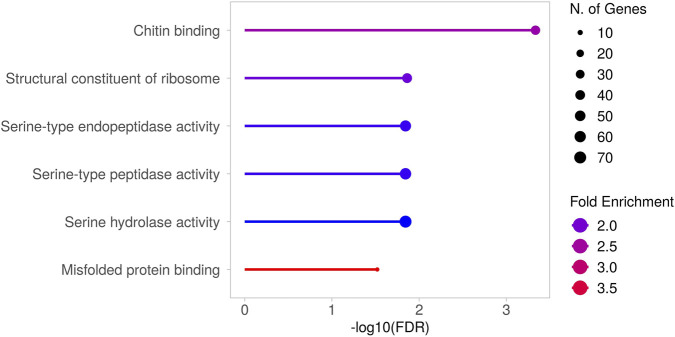
Gene Ontology (GO) analysis (MF) of differentially expressed genes in *TBPH* KO larvae. Results obtained from the GO analysis performed on the genes found to be differentially expressed between *TBPH* KO and control larvae. The significantly enriched molecular functions (MF) are depicted. Supplementary Figure S3 presents the heatmap showing the expression levels of the genes responsible for these enrichments. The complete list of enriched terms is provided in Supplementary Table S2.

Moreover, the GO analysis also revealed novel and hidden insights into ALS pathology, highlighting biological processes and molecular functions that remain largely unexplored or insufficiently characterized. The most interesting results were obtained by examining the enriched GO terms belonging to the Molecular Function (MF) category (Figure 2). While the term “Misfolded protein binding” is somewhat expected due to the well-known protein misfolding that occurs in the disease and leads to the formation of aggregated proteins and inclusions (Parakh and Atkin, 2016), the other terms underscore distinct and still emerging aspects of the pathology, as further elaborated in the Discussion.

### DEGs tissue distribution suggests a broader tissue involvement in the disease

**FIGURE 3:**
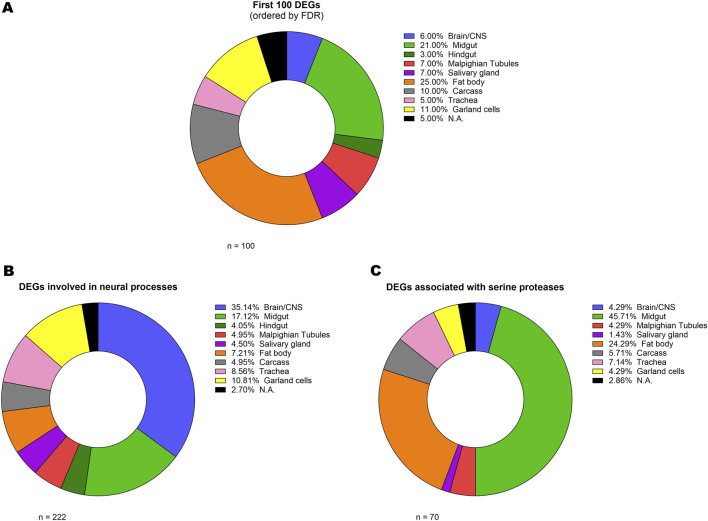
Analysis of gene tissue distribution. Donut charts representing the relative percentage of the main larval tissue of expression (basing on FlyAtlas 2 database) of the 100 most differentially expressed genes **(A)** and of the genes that, according to the GO analysis, were associated with neural pathways **(B)** and serine peptidase and hydrolase activity **(C)**. The complete list including all the genes and their respective Larval FPKM and Enrichment scores is provided in Supplementary Table S3.

While our primary objective was to get a molecular overview of the *TBPH* KO larval model in its entirety, we were also interested in understanding where the most relevant DEGs were mainly expressed, thus exploring the anatomical regions most implicated in the pathology. To do so, we took advantage of FlyAtlas 2 database (Krause et al., 2022), from which we obtained the main larval tissue of expression of all the analysed DEGs (Supplementary Table S3). Firstly, we performed this kind of analysis on the 100 most prominent DEGs in terms of FDR (Figure 3A; Supplementary Table S3). The results of this analysis revealed a wide distribution in terms of main tissue of expression, yet with a marked involvement of the fat body (25%) and the gut (24%, with 21% in the midgut and 3% in the hindgut). However, since this analysis could be influenced by the proportion of each tissue in the whole larva (*e*.*g*., the fat body lipid mass alone represents the 15% of the total body weight of third instar larvae (Baker and Thummel, 2007), we decided to integrate this approach with the data from the previous GO analysis. Specifically, we determined the primary tissue of expression of the genes that, according to the GO analysis, were associated with neuronal pathways (Figure 3B; Supplementary Table S3). While, as expected, the Brain/CNS was found to be the most enriched tissue (35%), the gut was confirmed as a relevant hub for the expression of these genes (21%, with 17% in the midgut and 4% in the hindgut). Interestingly, a similar result was obtained for the genes associated with serine peptidase and hydrolase activity (Figure 3C; Supplementary Table S3), with 45% of them being expressed in the midgut.

### TBPH depletion affects gene transcript usage

As part of the hnRNP family, TDP-43 is essential for regulating different aspects of RNA metabolism, particularly splicing, either directly or through the interaction with other hnRNPs (Ayala et al., 2005; Tollervey et al., 2011; Polymenidou et al., 2011; Buratti et al., 2005; Freibaum et al., 2010). Specifically, one of the main functions of TDP-43 - and of its *Drosophila* ortholog *TBPH* - is splicing repression (Donde et al., 2019), a process that is disrupted in ALS and FTD (Ling et al., 2015). Consistently, its depletion results in abnormal splicing events (Koike, 2024), and alterations in alternative splicing have been frequently reported in ALS patients (Miwa et al., 2025).

**FIGURE 4:**
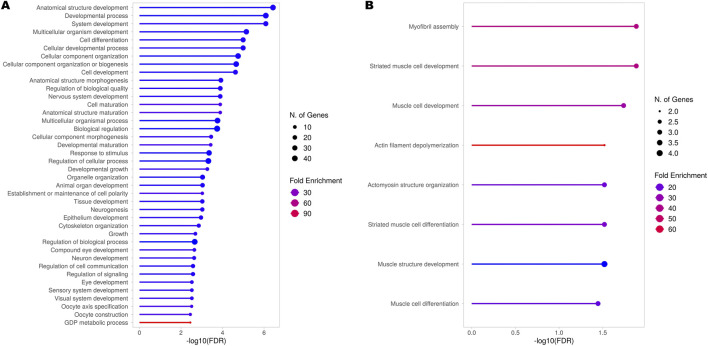
Gene Ontology (GO) analysis (BP) of genes showing Differential Transcript Usage (DTU). Results obtained from the GO analysis performed on all the genes identified by the RNA-seq experiment **(A)** and only the differentially expressed genes (DEGs) obtained from the comparison between *TBPH* KO and control larvae **(B)** exhibiting significant DTU across all detected transcripts. The top 40 or the complete set of significantly enriched biological processes (BP) are depicted. The complete lists of enriched terms are provided in Supplementary Tables S5 and S6, respectively.

Hence, even though our RNA-seq experiment did not allow for a complete analysis of all transcripts - mainly due to technical limitations given by 3′ RNA-seq protocols, providing limited coverage of full-length transcripts (see Methods) - we decided to at least preliminarily explore potential alternative transcript usage among the identified genes. With this analysis, we were able to identify 78 genes showing significant Differential Transcript Usage (DTU) (Supplementary Table S4). Subsequent GO analysis of these genes revealed their involvement in processes mainly associated with systemic and nervous system development (Figure 4A) but also including more specific terms associated with neuron projections, dendrite morphogenesis, axon extension, and synapse assembly (Supplementary Table S5). In addition, to investigate also genes in which alternative transcript usage might have led to changes in gene expression, we performed a GO analysis specifically on the subset of genes exhibiting DTU that were also differentially expressed between *TBPH* KO and control larvae (Figure 4B; Supplementary Table S6). Our results highlighted a clear involvement of these DEGs in processes almost exclusively associated with muscle cells development and differentiation.

## Discussion

### What we knew: identification of genes and pathways previously associated with ALS

As expected, the samples obtained from *TBPH* KO larvae clustered distinctly and exhibited a clear separation from those obtained from control larvae, resulting in a total of 1718 DEGs. Subsequent GO analysis revealed that a large proportion of these DEGs were involved in pathways related to neuronal development, morphogenesis, and physiology, and also to cell and in particular neuronal projections, which is consistent with the neurodegenerative phenotype associated with ALS pathology. Effectively, numerous genes taking part in the enriched neuronal processes were previously associated with ALS and in general with neurodegeneration, like *Atx2* and *Nedd4*. *Atx2*, a gene already associated with ALS, is essential for cytoskeletal dynamics and neurodevelopment; its depletion causes multiple morphological defects in the nervous system of third instar larvae, including impaired axon development and decreased dendrite outgrowth (Del Castillo et al., 2021). *Nedd4* encodes an E3 ubiquitin ligase that plays a key role in the ubiquitin-proteasome system of protein degradation and is therefore essential for the clearance of proteins that may accumulate and form toxic aggregates, as occurs in ALS (Haouari et al., 2022). In addition, we also reported the altered expression of genes not necessarily associated with the enriched neuronal pathways but known to play key roles in ALS pathogenesis, such as *Sod1* and *CHMP2B*. *Sod1*, which was found to be upregulated in *TBPH* KO larvae, was the first gene associated with both familial and sporadic forms of ALS and is the second most mutated gene in Caucasian patients, primarily through toxic gain-of-function mutations (Gagliardi et al., 2023; Benatar et al., 2025). *CHMP2B*, also found to be upregulated, was associated with ALS and FTD, with the latter having, among its causes, a gain-of-function mutation in the *CHMP2B* gene (Ugbode and West, 2021; Chen et al., 2022).

### What is new: further insights into ALS pathology

The GO analysis also revealed the enrichment of terms associated with molecular functions that have only recently emerged in the context of ALS, or that have long remained unexplored or insufficiently characterized. For example, the genes underlying the enrichment of the term “Chitin binding” include *Idgf1*, *Idgf3*, and *Idgf4*, which encode chitinase-like proteins (Sustar et al., 2023). Interestingly, chitinases and chitinase-like proteins are recently emerging as a potential diagnostic and prognostic biomarker for neurologic disorders (Pinteac et al., 2020), and in particular for ALS (Xu et al., 2024). As for “Structural constituent of ribosome”, disruptions in ribosome function are increasingly recognized as key contributors to the disease’s progression (Lehmkuhl et al., 2021; Loveland et al., 2022). Moreover, one of the most noteworthy observations is the marked enrichment of terms associated with serine proteases/hydrolases. Serine proteases are a class of enzymes that catalyse the hydrolysis of peptide bonds, characterized by the presence of a serine residue within their active site. Although best known for their roles in coagulation and digestion, several serine proteases are also expressed in the brain, where they play important roles in the development, physiology, and pathophysiology of the nervous system (Wang et al., 2008; Almonte and Sweatt, 2011). To date, regarding ALS, little is known about the roles of serine proteases in the disease. However, a study reported the presence of three serine proteases (trypsin, chymotrypsin, and thrombin) in neurofilaments conglomerates, which are histopathological hallmarks of early-stage ALS, within motor neurons of ALS patients (Chou et al., 1998). Moreover, plasminogen and tissue plasminogen activator (tPA) intraperitoneal administration can induce motor neurons degeneration in mice (Demestre et al., 2006), and urokinase-type plasminogen activator (uPA) exhibits higher expression in the ventral horn of the spinal cord of G93A *SOD1* mice and ALS patients (Glas et al., 2007).

### DEGs tissue distribution revealed a possible hidden role for the gut

To investigate the anatomical regions most relevant to the pathology, we identified the main larval tissue in which selected subsets of DEGs were expressed by using FlyAtlas 2 (Krause et al., 2022). For DEGs associated with neuronal pathways, the Brain/CNS was, as expected, the most enriched tissue (35%); however, notably, also the gut emerged as a relevant hub for their expression (21%), possibly highlighting, also in this model, the contribution of the gut and enteric nervous system in the pathophysiology of ALS, as previously reported in several studies (Lee et al., 2024; Zhang et al., 2021; Luesma et al., 2024). This is of particular interest given that the fruit fly has proven to be an ideal model for investigating the gut-brain axis in the context of metabolic and neurodegenerative diseases (Sadaqat et al., 2022; Kitani-Morii et al., 2021). Interestingly, a marked involvement of the gut (45%) also emerged from the analysis of DEGs associated with serine proteases, which are capable of exciting myenteric neurons through protease-activated receptors (Gao et al., 2002).

### Effects of TBPH depletion on transcript usage corroborate its elusive role in muscles

In light of TDP-43 essential role in regulating splicing (Ayala et al., 2005; Tollervey et al., 2011; Donde et al., 2019), we preliminarily explored potential DTU among the genes identified by our RNA-seq experiment. The GO analysis of these genes highlighted their roles predominantly in systemic and nervous system development, but also in more specific processes such as neuron projection development, dendrite morphogenesis, axon extension, and synapse assembly. Interestingly, this group included genes such as *CadN*, which encodes an important hub molecule implicated in neurodevelopmental and neurodegenerative diseases (László and Lele, 2022), and *Rab5*, whose encoded protein was shown to accumulate in the cytoplasm of spinal cord neurons of patients with ALS (Sanhueza et al., 2015). Moreover, the GO analysis of genes exhibiting both DTU and differential expression between *TBPH* KO and control larvae pointed to a clear involvement of these genes in processes associated with muscle cells development and differentiation, supporting and extending recent findings in the field of TDP-43 pathology. Indeed, after the evidence that TDP-43 was essential for normal skeletal muscle formation by assembling cytoplasmic amyloid-like structures called myo-granules (Vogler et al., 2018), which gave rise to the discussion about its beneficial or detrimental role in muscle cells physiology and pathology (McHugh, 2019), several works have tried to uncover its specific role in this tissue (Šušnjar et al., 2022; Versluys et al., 2022) and also its possible implications in the context of ALS (Versluys et al., 2022; Cykowski et al., 2018; Mori et al., 2019).

## Conclusion

Overall, this study provides the first comprehensive molecular characterization of whole third instar *TBPH* ^Δ23/Δ23^ larvae, one of the most important *Drosophila melanogaster* models for *TDP-43*-mediated ALS. We first reported how the gene expression alterations occurring in *TBPH* KO larvae are consistent with those expected in this pathological context, thereby validating and further supporting its relevance as a model even at the transcriptomic level. We also identified several DEGs involved in novel or still poorly characterized biological processes and molecular functions linked to ALS, with a particular emphasis on serine proteases/hydrolases metabolism. Furthermore, we explored the involvement of tissues beyond the nervous system in ALS pathogenesis - most notably, the gut, which emerged as primary site of expression for a number of genes of interest, and the muscles, since DEGs with DTU were almost exclusively associated with muscle cell development and differentiation. Such systemic inside underscores the increased appreciation of ALS as more than a motoneuron disease, with this work providing new insights that support a whole-organism, multisystemic view of the pathological mechanisms of ALS, thus opening new pathways for drug discovery.

## Data Availability

The datasets presented in this study can be found in online repositories. The names of the repository/repositories and accession number(s) can be found below: https://www.ncbi.nlm.nih.gov/, PRJNA1289126.
