# Supplementary figures and images for: TDP-43-mediated amyotrophic lateral sclerosis: new/hidden insights from Drosophila
Source: Front Cell Dev Biol. 2025 Oct 16;13:1677090. doi: 10.3389/fcell.2025.1677090 (PMC12573270; doi:10.3389/fcell.2025.1677090)

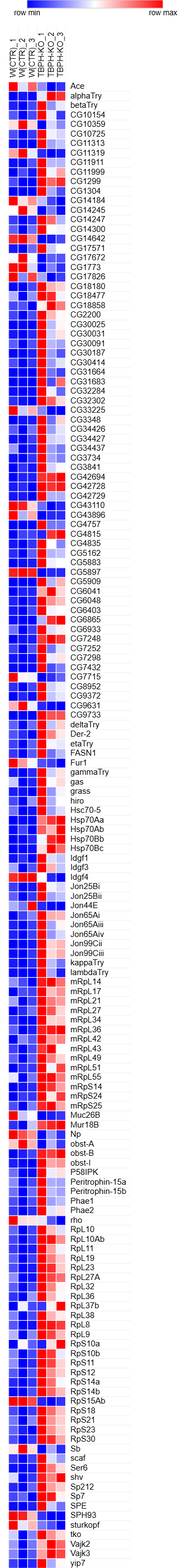

Supplement: Supplementary file 3 [file Image3.tif]

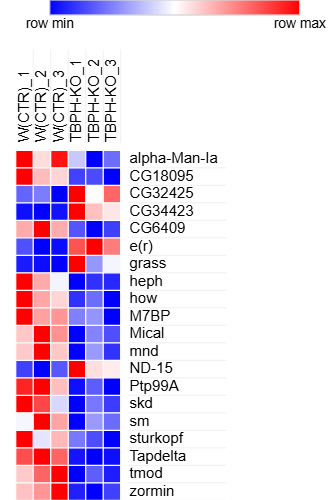

Supplement: Supplementary file 4 [file Image4.tif]

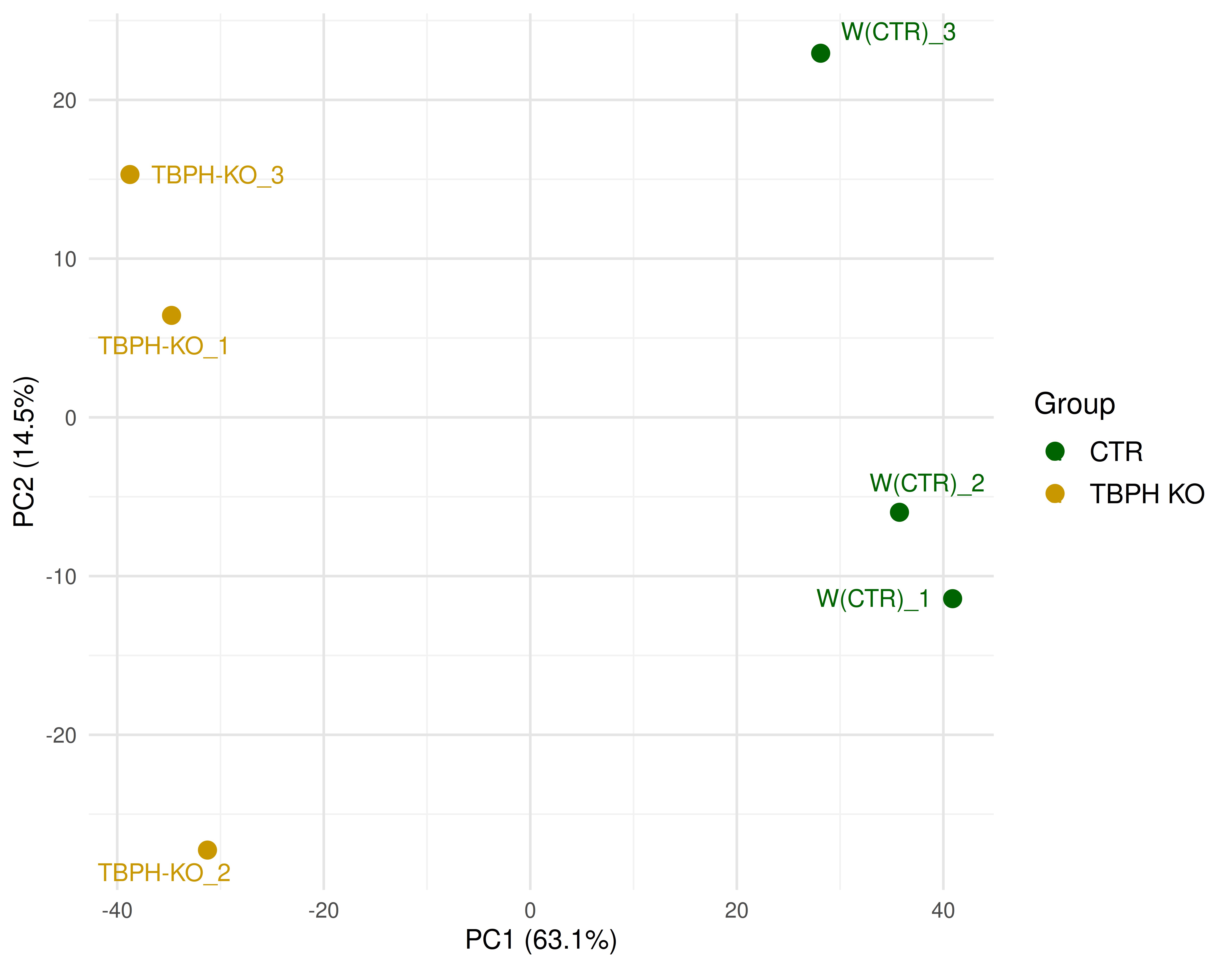

Supplement: Supplementary file 5 [file Image1.jpeg]

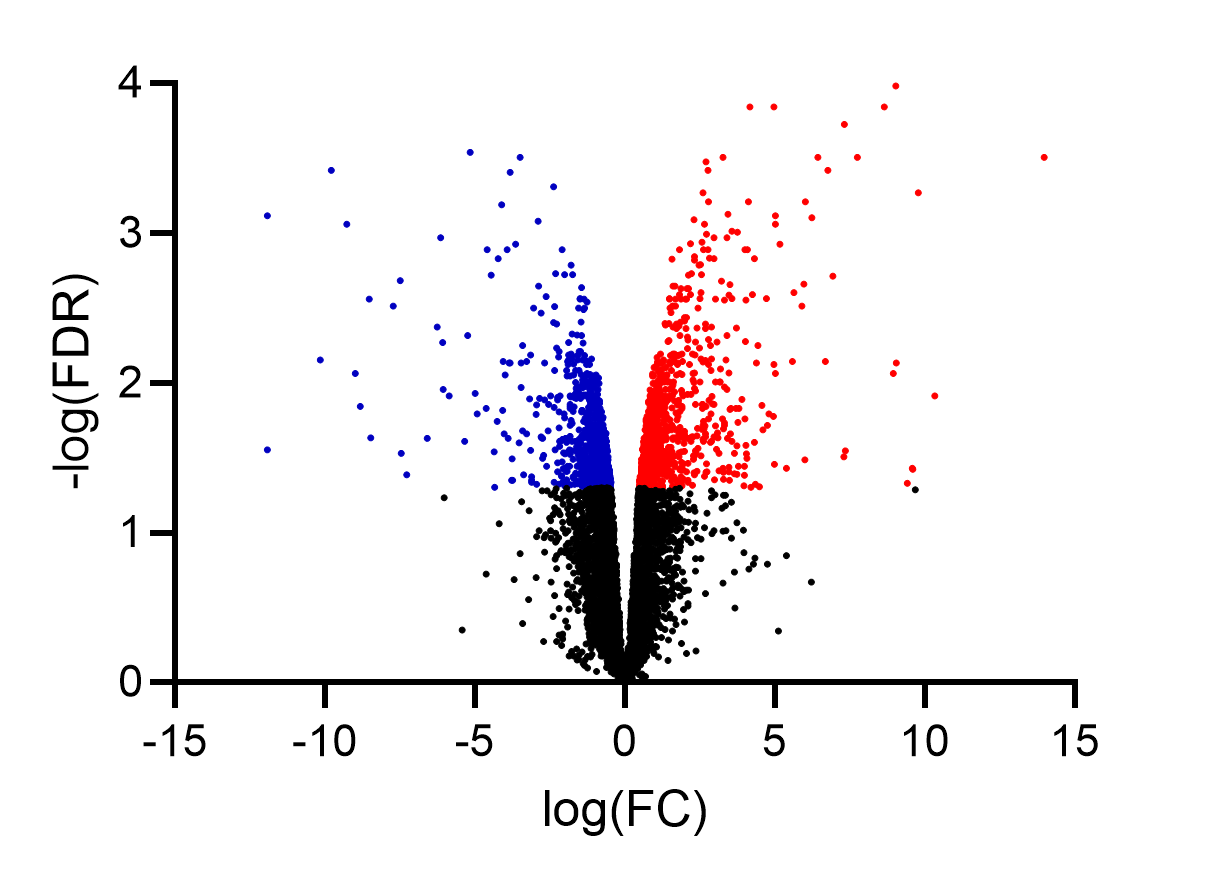

Supplement: Supplementary file 6 [file Image2.tif]
