# Supplementary material for: TDP-43-mediated amyotrophic lateral sclerosis: new/hidden insights from Drosophila
Source: Front Cell Dev Biol. 2025 Oct 16;13:1677090. doi: 10.3389/fcell.2025.1677090 (PMC12573270; doi:10.3389/fcell.2025.1677090)
Supplement: Supplementary file 11 [file DataSheet1.docx]

**SUPPLEMENTARY MATERIALS**

**Figure S1. Principal Component Analysis (PCA) of *TBPH* KO and control whole larvae samples.** PCA was performed on gene expression data obtained from RNA-seq analysis of total RNA extracted from *TBPH* KO (in yellow) and control (in green) whole larvae.

**Figure S2. Volcano plot of differential gene expression between *TBPH* KO and control whole larvae.** Volcano plot showing differentially expressed genes (DEGs) identified by RNA-seq analysis of total RNA extracted from *TBPH* KO and control whole third instar larvae. Out of 1718 DEGs (identified at FDR < 0.05), 845 genes were down-regulated (in blue) and 873 were up-regulated (in red) in *TBPH* KO *vs*. control larvae (Table S1).

**Figure S3. Heatmap of the genes driving the enrichment of the Gene Ontology (GO) terms in Figure 2.** Heatmap showing the expression levels in *TBPH* KO and control whole third instar larvae of the genes responsible for the enrichment of the GO terms presented in Figure 2.

**Figure S4. Heatmap of differentially expressed genes (DEGs) showing Differential Transcript Usage (DTU).** Heatmap showing the expression levels in *TBPH* KO and control whole third instar larvae of the genes found to be differentially expressed (Table S1) and showing DTU (Table S4).

**Table S1. List of differentially expressed genes (DEGs) between *TBPH* KO and control whole larvae.**List of DEGs identified by RNA-seq analysis of total RNA extracted from *TBPH* KO and control whole third instar larvae. While only genes with a false discovery rate (FDR) < 0.05 were considered statistically significant and used for downstream analyses, in order to provide a broader overview this list also includes genes with FDR < 0.1.

**Table S2. List of gene ontology (GO) enriched terms obtained from the DEGs between *TBPH* KO and control whole larvae.** List of biological processes (BP), cellular components (CC), and molecular functions (MF) which were significantly enriched (FDR < 0.05) in the GO analysis of the DEGs obtained from the comparison between *TBPH* KO and control whole third instar larvae.

**Table S3. List of DEGs for which the main tissue of expression was analysed and corresponding scores.**List of specific subsets of DEGs for which the main larval tissue of expression was identified from FlyAtlas 2 (flyatlas2.org), including the Larval FPKM and Enrichment scores for each larval tissue.

**Table S4. List of genes showing Differential Transcript Usage (DTU).** List of genes identified by the
RNA-seq experiment exhibiting significant (adjusted p-value < 0.05) DTU across all detected transcripts: a total of 78 genes passed the stageR screen and were considered to show DTU, of which 20 were also differentially expressed between *TBPH* KO and control samples.

**Table S5. List of GO enriched terms obtained from all the genes showing DTU.** List of biological
processes (BP), cellular components (CC), and molecular functions (MF) which were significantly enriched (FDR < 0.05) in the GO analysis of all the genes identified by the RNA-seq experiment exhibiting DTU.

**Table S6. List of GO enriched terms obtained from the DEGs showing DTU.** List of biological
processes (BP) and cellular components (CC) which were significantly enriched (FDR < 0.05) in the GO analysis of the DEGs obtained from the comparison between *TBPH* KO and control larvae exhibiting DTU.
